# The hemispheric contrast in cloud microphysical properties constrains aerosol forcing

**DOI:** 10.1073/pnas.1922502117

**Published:** 2020-07-27

**Authors:** Isabel L. McCoy, Daniel T. McCoy, Robert Wood, Leighton Regayre, Duncan Watson-Parris, Daniel P. Grosvenor, Jane P. Mulcahy, Yongxiang Hu, Frida A.-M. Bender, Paul R. Field, Kenneth S. Carslaw, Hamish Gordon

**Affiliations:** ^a^Atmospheric Sciences Department, University of Washington, Seattle, WA 98105;; ^b^Institute for Climate and Atmospheric Science, School of Earth and Environment, University of Leeds, LS2 9JT Leeds, United Kingdom;; ^c^Department of Physics, University of Oxford, OX1 3PU Oxford, United Kingdom;; ^d^National Center for Atmospheric Science, University of Leeds, LS2 9JT Leeds, United Kingdom;; ^e^Met Office, Exeter EX1 3PB, United Kingdom;; ^f^Atmospheric Composition Branch, NASA Langley Research Center, Hampton, VA 23681;; ^g^Department of Meteorology, Stockholm University, SE-106 91 Stockholm, Sweden;; ^h^Bolin Centre for Climate Research, Stockholm University, SE-106 91 Stockholm, Sweden;; ^i^College of Engineering, Carnegie-Mellon University, Pittsburgh, PA 15213

**Keywords:** cloud droplet number concentration, radiative forcing, aerosol−cloud interactions, Southern Ocean, remote sensing

## Abstract

The change in planetary albedo due to aerosol−cloud interactions during the industrial era is the leading source of uncertainty in inferring Earth’s climate sensitivity to increased greenhouse gases from the historical record. The variable that controls aerosol−cloud interactions in warm clouds is droplet number concentration. Global climate models demonstrate that the present-day hemispheric contrast in cloud droplet number concentration between the pristine Southern Hemisphere and the polluted Northern Hemisphere oceans can be used as a proxy for anthropogenically driven change in cloud droplet number concentration. Remotely sensed estimates constrain this change in droplet number concentration to be between 8 cm^−3^ and 24 cm^−3^. By extension, the radiative forcing since 1850 from aerosol−cloud interactions is constrained to be −1.2 W⋅m^−2^ to −0.6 W⋅m^−2^. The robustness of this constraint depends upon the assumption that pristine Southern Ocean droplet number concentration is a suitable proxy for preindustrial concentrations. Droplet number concentrations calculated from satellite data over the Southern Ocean are high in austral summer. Near Antarctica, they reach values typical of Northern Hemisphere polluted outflows. These concentrations are found to agree with several in situ datasets. In contrast, climate models show systematic underpredictions of cloud droplet number concentration across the Southern Ocean. Near Antarctica, where precipitation sinks of aerosol are small, the underestimation by climate models is particularly large. This motivates the need for detailed process studies of aerosol production and aerosol−cloud interactions in pristine environments. The hemispheric difference in satellite estimated cloud droplet number concentration implies preindustrial aerosol concentrations were higher than estimated by most models.

## Significance

Enhancement of aerosol that can nucleate cloud droplets increases the droplet number concentration and albedo of clouds. This increases the amount of sunlight reflected to space. Uncertainty in how aerosol−cloud interactions over the industrial period have increased planetary albedo by this mechanism leads to significant uncertainty in climate projections. Our work presents a method for observationally constraining the change in albedo due to anthropogenic aerosol emissions: a hemispheric difference in remotely sensed cloud droplet number between the pristine Southern Ocean (a preindustrial proxy) and the polluted Northern Hemisphere. Application of this constraint to climate models reduces the range of estimated albedo change since industrialization and suggests current models underpredict cloud droplet number concentration in the preindustrial era.

The change in reflected shortwave radiation between the preindustrial (PI) and the present day (PD) due to anthropogenic emissions of aerosols, known as aerosol radiative forcing, is the leading cause of uncertainty in inferring climate sensitivity from the observational record (1, 2). A recent survey identified the dominant contributor to the uncertainty in global mean aerosol radiative forcing as aerosol−cloud interactions (aci) in liquid clouds (3). Aerosols change the radiation reflected back to space by liquid clouds in two ways: 1) by modulating the number concentration of cloud droplets (*N*_*d*_), which changes cloud reflectivity even without any changes to cloud macrostructure (4); 2) by changing *N*_*d*_, cloud microphysical processes are altered that have various impacts on cloud macrophysical properties [e.g., cloud cover or liquid water content (5)]. These effects are referred to as radiative forcing due to aci (*RF*_*aci*_) and cloud adjustments to aerosol, respectively (6). The combined forcing from aerosol−cloud adjustments and *RF*_*aci*_ is referred to as the effective *RF*_*aci*_ (*ERF*_*aci*_). The net aerosol forcing is the sum of *ERF*_*aci*_ and a similar quantity for aerosol direct interactions, *ERF*_*ari*_. Here, we focus on providing an observational constraint for the change in *N*_*d*_ and *RF*_*aci*_. The forcing due to aerosol−cloud adjustments is uncertain in both sign and magnitude (7–13), but is expected to scale with changes in *N*_*d*_ (3). Narrowing the possible range of changes in *N*_*d*_ and resulting *RF*_*aci*_ will narrow uncertainty in *ERF*_*aci*_ and, by extension, improve our inference of climate sensitivity (1, 3).

*RF*_*aci*_ is nonlinearly dependent on the change in *N*_*d*_ over the industrial period (14). Natural aerosols, or aerosols in the PI state, are the largest cause of uncertainty in aerosol forcing over the industrial period (14, 15). The PD *N*_*d*_ is observable, but we must infer PI *N*_*d*_ using other means. Here, we use the pristine Southern Hemisphere (SH) (16) as a proxy for the PI and examine the contrast between the SH and the polluted Northern Hemisphere (NH) to estimate the anthropogenic perturbation to *N*_*d*_.

Previous studies have discussed the hemispheric contrast in cloud properties created by anthropogenic aerosol emissions in the NH. The effective radius of droplets (*r*_*e*_) is smaller in the NH than in the SH (17, 18). Feng and Ramanathan (18) found that a chemical transport model driven by reanalysis meteorology was able to produce a difference in *N*_*d*_ between the NH and SH consistent with hemispheric contrasts in satellite retrievals of *r*_*e*_ and cloud optical depth (*τ*). Boucher and Lohmann (19) used the hemispheric difference in *r*_*e*_ to evaluate the robustness of the *RF*_*aci*_ simulated in instances of the LMD (Laboratoire de Météorologie Dynamique) and ECHAM (European Center for Medium-range Weather Forecasting, Hamburg version) global climate models (GCMs) when a prescribed relationship between sulfate mass and *N*_*d*_ was implemented. As in these pioneering works, we use hemispheric differences in cloud microphysics to evaluate modeled aci. Our approach differs from previous work in the following ways. First, *r*_*e*_, while readily retrieved by remote sensing, is a function of both the number concentration of cloud condensation nuclei (CCN) and the liquid water content of clouds. The differences in cloud liquid water content between hemispheres (18, 20) will weaken any *r*_*e*_-based constraint on hemispheric CCN difference. *N*_*d*_ is calculated from remote sensing retrievals of both *r*_*e*_ and *τ*, which helps to account for cloud liquid water contributions as outlined in Grosvenor et al. (21). We use this calculated *N*_*d*_ to constrain *RF*_*aci*_ because it is the key variable linking cloud microphysical and aerosol properties (22). Second, we analyze output from a large collection of GCMs designed to quantify aerosol forcing alongside a million-member ensemble from a single model that samples uncertainty in 26 aerosol processes (23). This enables us to robustly quantify and then constrain the uncertainty in the change in *N*_*d*_ and *RF*_*aci*_.

The *N*_*d*_ derived from satellite retrievals has been shown to be reasonably unbiased in comparison with aircraft measurements (21, 24–28) and to agree well in both the remote Southern Ocean (SO) (29) and the NH (28). Biases between in situ and *N*_*d*_ calculated based on Moderate Resolution Spectroradiometer (MODIS) data are on the order of 1 cm^−3^ to 20 cm^−3^, depending on geographic region and boundary layer stratification, and systematic bias does not scale strongly with *N*_*d*_ (27–29). The hemispheric contrast in *N*_*d*_ is a difference, so this should moderate the effects of any systematic biases in *N*_*d*_. Our understanding of the relationship between hemispheric contrast in *N*_*d*_ and anthropogenic perturbations to *N*_*d*_ is facilitated by insight into the uncertainty in the PI atmosphere provided by GCMs. We combine analysis of structural model uncertainty from CMIP5 models participating in the Aerocom phase II project (30) and several simulations made during the development of the atmosphere-only climate model configuration, HadGEM3-GA7.1 (Hadley Center Global Environmental Model) (31) with analysis of parametric uncertainty within a perturbed parameter ensemble (PPE) in HadGEM3-GA4-UKCA (United Kingdom Chemistry and Aerosols). The PPE is based on 235 individual simulations in which combinations of 26 aerosol processes and emissions were perturbed (23). The output from these 235 simulations was used to train Gaussian process emulators to enable a million model variants to be generated, facilitating more robust statistical analysis (32). We show that uniting this growing confidence in satellite-derived *N*_*d*_ with state-of-the-art modeling experiments directed at evaluating aci in warm clouds allows us to bound anthropogenic perturbations to *N*_*d*_ and *RF*_*aci*_ over the industrial period.

## Results

### Definition and Application of a Hemispheric Contrast.

**Fig. 1.**
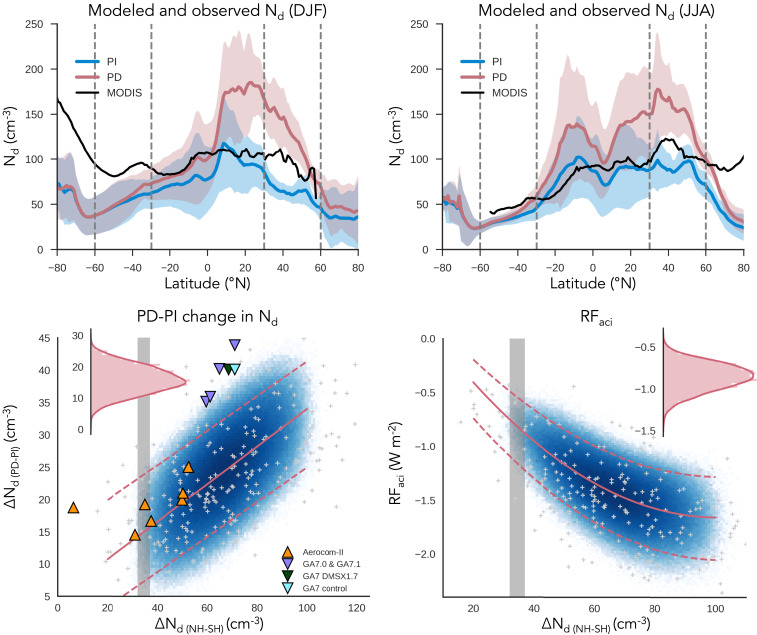
Constraints on aci from satellite estimated hemispheric contrast in *N*_*d*_ over oceans (∆*N*_*d(NH-SH)*_). (*A* and *B*) Oceanic PI (blue) and PD (red) *N*_*d*_ modeled by Aerocom-II models and HadGEM3-GA7.1 development models. Thick lines show the multimodel mean, and corresponding shading shows the SD across models. (*A* and *B*) Data from (*A*) December through February (DJF) and (*B*) June through August (JJA). In SO winter, the Aerocom-II National Center for Atmospheric Research models are missing data due to lack of low, liquid cloud, leading to discontinuity in the multimodel mean at 70°S. Zonal means from each model are shown in *SI Appendix*, Fig. S1. ∆*N*_*d(NH-SH)*_ is calculated as the difference in annual, area-weighted mean *N*_*d*_ over the ocean between 30°N to 60°N and 30°S to 60°S (averaging boundaries shown as vertical dashed lines). (*C*) Change in oceanic *N*_*d*_ between the PI and PD (∆*N*_*d(PD-PI)*_) as a function of ∆*N*_*d(NH-SH)*_ in PPE members (gray crosses for individual model members, blue shading for *N*_*d*_ values sampled from a statistical emulator), in Aerocom-II (orange triangles), and HadGEM-GA7.1 development models (purple, blue, and dark green triangles). HadGEM-GA7.0 with enhanced DMS is shown in dark green and the control HadGEM-GA7.0 in blue. The linear fit to the PPE data and 95% prediction bands on the fit are shown as red solid and dashed lines. The 95% confidence on the interannual range of *∆N*_*d(NH-SH)*_ estimated by MODIS is shown in gray. (*D*) As in *C* but showing the relation between *RF*_*aci*_ and the hemispheric contrast calculated from the PPE sample members along with a second-order polynomial fit between ∆*N*_*d(NH-SH)*_ and *RF*_*aci*_. (*Insets*) The PDF of the emulated PPE member values within the observationally constrained range of ∆*N*_*d(NH-SH)*_ (*C*) for ∆*N*_*d(PD-PI)*_ and (*D*) for *RF*_*aci*_.

**Fig. 2.**
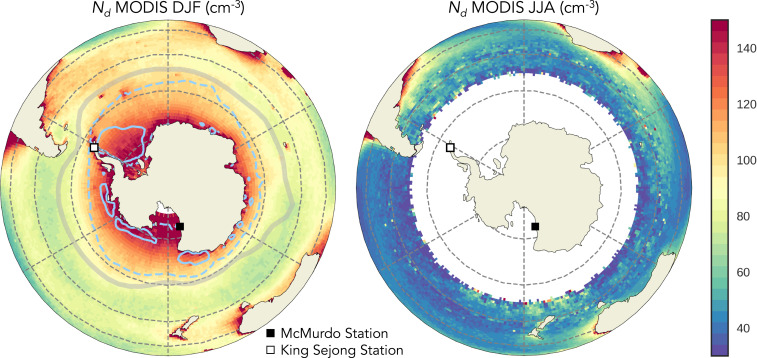
Mean *N*_*d*_ calculated from MODIS data in (*A*) summer (DJF) and (*B*) winter (JJA). Seasonal mean sea ice contours from OSTIA fractional sea ice are shown as dashed (1%) and solid blue lines (50%). Locations are shown for McMurdo Station (48) (solid square) and King Sejong Station (47) (empty square). The position of the DJF lower tropospheric storm track (74) is shown with a gray line.

Comparing satellite-derived, maritime *N*_*d*_ from MODIS with Aerocom phase II and HadGEM3-GA7.1 development simulations reveals major discrepancies between GCMs and MODIS *N*_*d*_ in the PD. GCMs consistently overestimate tropical and NH midlatitude *N*_*d*_ (Fig. 1 *A* and *B*). They consistently underestimate summertime *N*_*d*_ in the SH midlatitude (30°S to 60°S) (Fig. 1*A*). GCMs also underestimate summertime marine *N*_*d*_ poleward of 60° in both hemispheres, especially in the SH, where MODIS *N*_*d*_ increases significantly toward Antarctica (Figs. 1*A* and 2*A*). Intriguingly, the mean MODIS summertime *N*_*d*_ near Antarctica is close to values found in continental outflows from heavily industrialized regions (28). The remote SO is among the most pristine regions in the world (16), with emissions from ocean biology controlling aerosol and *N*_*d*_ seasonality (33, 34). The NH midlatitude has both polluted and pristine aerosol influences. The magnitude of the summertime Arctic MODIS *N*_*d*_ increase is smaller than the summertime Antarctic increase, possibly due to the closer proximity to large continental and anthropogenic sources of aerosol in the NH [and the nonlinear relationship between CCN and *N*_*d*_ (14)]. While more complex to disentangle, the natural sources of NH aerosol have a significant seasonal cycle driven by ocean biology (35–37). These high summertime midlatitude and high-latitude satellite-derived *N*_*d *_values in the SH and high-latitude satellite-derived *N*_*d *_values in the NH are not captured by GCMs but, as discussed below, are supported by in situ observations of CCN and *N*_*d*_. PPE model members show similar discrepancies compared with MODIS, although NH values are less overestimated, on average (*SI Appendix*, Fig. S2).

As there are no observations of PI *N*_*d*_, the accuracy of modeled PI *N*_*d*_ cannot be evaluated directly. However, we are able to draw three qualitative conclusions regarding the PI and PD *N*_*d*_ from the models. First, in the GCMs, the majority of the PD−PI change is in the NH. This is consistent with the zones of maximum anthropogenic emissions and direct aerosol forcing (18, 38). Second, sources of CCN over the SO are largely marine with very small contributions from continents, and levels that are mostly unchanged from the PI to the PD (16). Third, our analysis of the Aerocom phase II and HadGEM3-GA7.1 development simulations show the PI *N*_*d*_ is fairly similar in the NH and SH, with a difference in the 30°- to 60°-latitude bands over oceans for these simulations of 16 ± 7 cm^−3^ at 95% confidence. In contrast, simulated PD *N*_*d*_ difference between these bands is 43 ± 8 cm^−3^ at 95% confidence. The larger PI *N*_*d*_ in the NH compared to the SH is primarily due to biomass burning emissions in the NH (39). However, the relative hemispheric symmetry in PI *N*_*d*_ is consistent with modeling studies of aerosol sources over oceans in the PI, where marine sources contribute a large fraction of marine CCN in both hemispheres (40, 41).

Based on our ability to estimate *N*_*d*_ in the PD from remote sensing retrievals and the aforementioned inferences from GCMs, we can use the hemispheric PD *N*_*d*_ difference between the polluted NH and the pristine SH oceans to gain insight into the change in global mean area-weighted *N*_*d*_ between PD and PI over land and ocean (*∆N*_*d(PD-PI)*_). We find that there is a positive correlation between *∆N*_*d(PD-PI)*_ and the differences in marine *N*_*d*_ between 30°N to 60°N and 30°S to 60°S (∆*N*_*d(NH-SH)*_) within the various GCMs examined in this study and the members of the PPE (Fig. 1*C*). Examination of the million-member sample shows that ∆*N*_*d(NH-SH)*_ is approximately linearly correlated with *∆N*_*d(PD-PI)*_ (*R*^2^ = 0.3). The Aerocom phase II models fall within the 95% prediction interval of the best fit to the PPE sample members, except for ECHAM6. This may be due to ECHAM6’s imposed minimum *N*_*d*_ of 40 cm^−3^, which is near the mean PI *N*_*d*_ in the GCMs surveyed here (Fig. 1 *A* and *B*). MODIS estimates ∆*N*_*d(NH-SH)*_ to be between 32 cm^−3^ and 37 cm^−3^ with 95% confidence. To agree with the satellite estimated range and the linear fit to the PPE, *∆N*_*d(PD-PI)*_ is predicted to be 8 cm^−3^ to 24 cm^−3^ at 95% confidence (Fig. 1*C*).

The *∆N*_*d(PD-PI)*_ predicted by the HadGEM3-GA7.1 development models is on the upper end of what is predicted by the PPE. This is consistent with the stronger *ERF*_*aci*_ in GA7 model versions (−2.75 W⋅m^−2^ in GA7.0, −1.45 W⋅m^−2^ in GA7.1) compared to the weaker aerosol forcing in the GA4.0 model version used in the PPE (−1.51 Wm^−2^ on average with a 95% credible range of −2.04 W⋅m^−2^ to −0.96 W⋅m^−2^) (23, 42). The spread of *∆N*_*d(PD-PI)*_ between the PPE, Aerocom, and HadGEM3-GA7 models demonstrates the importance of examining multiple GCMs to consider structural differences. Because few of the GCMs (three of the eight Aerocom models and none of the HadGEM3-GA7 models) are consistent with MODIS *N*_*d*_, we also demonstrate the usefulness of sampling uncertainty within a single model by using the PPE. Using the million-member sample helps us to avoid the equifinality issues raised by examining a single model variant (43, 44) and produces a small subset of model variants within the observational range. Further investigation of the aerosol parameters important in this subset of member variants may help us to understand the processes that are key to producing values of *N*_*d*_ that are consistent with satellite data.

We have constrained changes in *∆N*_*d(PD-PI)*_ using satellite estimated ∆*N*_*d(NH-SH)*_. A similar constraint can be applied to *RF*_*aci*_. The Aerocom phase II and HadGEM3-GA7.1 development models include aerosol−cloud adjustments, so, for this analysis, we rely on our million-member sample from the PPE which has no aerosol−cloud adjustments. We find that the *RF*_*aci*_ is negatively correlated with ∆*N*_*d(NH-SH)*_ (Fig. 1*D*). We fit the relationship between *RF*_*aci*_ and ∆*N*_*d(NH-SH)*_ in the million-member sample using a second-order polynomial. For large values of ∆*N*_*d(NH-SH)*_, the spread in *RF*_*aci*_ from the PPE is very broad. However, when *N*_*d*_ is more symmetric between hemispheres, the range of *RF*_*aci*_ produced by different members of the PPE narrows. The prediction interval of the fit combined with the satellite estimated ∆*N*_*d(NH-SH)*_ constrains *RF*_*aci*_ to be between −1.2 W⋅m^−2^ and −0.6 W⋅m^−2^ at 95% confidence.

One caveat to our constraint on *RF*_*aci*_ and *∆N*_*d(PD-PI)*_ is that our methodology may suffer from the same limitations that all single-observation constraints suffer from, which is producing an overly tight constraint (45). However, combining this methodology with other observational constraints may avoid these single observation issues [e.g., as in the multiobservation constraint on *ERF*_*aci*_ in Johnson et al. (45) and Regayre et al. (46)] as well as help to constrain uncertainty associated with other processes in the models not captured by ∆*N*_*d(NH-SH)*_ (e.g., the aerosol optical depth constraint on *RF*_*ari*_ presented in ref. 32). We have chosen to only use satellite data over oceans in calculating ∆*N*_*d(NH-SH)*_ because it is more extensively evaluated against aircraft measurements (21). This broadens the constraint on global mean PI to PD changes in cloud properties (*∆N*_*d(PD-PI)*_ and *RF*_*aci*_ in Fig. 1 *C* and *D*, *Insets*) by ignoring information from satellite data over land. However, we choose to use more robust satellite data at the cost of a broader, but more reliable, constraint on global mean changes.

### Evaluating SO *N*_*d*_.

The hemispheric constraint depends on the accuracy of the satellite-derived values of PD *N*_*d*_. As noted in the Introduction, MODIS *N*_*d*_ has been extensively validated against aircraft measurements in the NH and parts of the SH (21, 28, 29). Because aircraft observations of *N*_*d*_ are not as plentiful in the more remote regions of the SO, we use other datasets to provide additional assessment of the quality and the believability of the surprising SO MODIS *N*_*d*_ pattern (Fig. 2).

The latitudinal and seasonal patterns of MODIS *N*_*d*_ are supported by the multiyear records of CCN from Antarctic ground sites at King Sejong Station (62°S) (47) and McMurdo Station (77°S) (48) (Fig. 2 and *SI Appendix*, Fig. S3*A*). Comparison of MODIS *N*_*d*_ within 4° of each station to the CCN station data shows matching summertime peaks and an increase with poleward latitude between King Sejong and McMurdo (*SI Appendix*, Fig. S3*A*). Summertime CCN measured at King Sejong was classified as largely biogenic (49, 50). This is consistent with measurements taken during cruises in the SO that observed increases in CCN, biogenic CCN precursor gases [i.e., phytoplankton emissions of dimethyl sulfide (DMS) which can be oxidized in the atmosphere to form either sulfate, an efficient CCN (19, 33, 51–54), or precursors for particle nucleation] (55), and concentrations of small particles that grow into CCN [i.e., nucleation-mode aerosols, often newly formed from biogenic precursor gases (56, 57)] (58) near Antarctica. The RITS (Radiatively Important Trace Species) campaign (59, 60) also observed a summertime increase in total aerosol concentration (including nucleation and CCN size aerosols) between its winter 1993 and summer 1994 cruises along the coast of Antarctica (*SI Appendix*, Fig. S3 map and *SI Appendix*, Fig. S3*B*).

We suspect that the summertime peak in *N*_*d*_ near Antarctica is linked to increases in biological activity as sea ice retreats in these regions. The seasonal sea ice zone’s high productivity and frequent, large phytoplankton blooms (61–63) lead to enhanced emissions of biogenic CCN precursor gases. Recent observations found increased concentrations of trace gases associated with DMS oxidation in the seasonal sea ice zone (55), nucleation-mode particles at ice edge (64), and nucleation-mode particles in biologically active basins near Antarctica (49, 50). The 2016 ORCAS (O_2_/N_2_ Ratio and CO_2_ Airborne Southern Ocean) flight campaign sampled *N*_d_ over both open water and broken ice in the seasonal sea ice zone in the Amundsen and Weddell Seas. ORCAS observed higher *N*_d_ over marginal sea ice than over open water [based on estimates of sea ice fraction (65) interpolated to the flight track; *SI Appendix*, Fig. S3*C*]. The high *N*_d_ over marginal sea ice observed during ORCAS (median of ∼140 cm^−3^) is consistent with the *N*_*d*_ observed during the OFCAP (Orographic Flow and the Climate of the Antarctic Peninsula) flights across the Antarctic peninsula (66) (*SI Appendix*, Fig. S3*C*) and with the MODIS *N*_*d*_. The increase in *N*_*d*_ over regions with marginal sea ice is also supported by MODIS *N*_*d*_ over the period 2003–2015 sampled along the ORCAS flight track (*SI Appendix*, Fig. S3*D*). Examination of the entire SO region by MODIS shows that, as the sea ice begins to retreat (October to November), *N*_*d*_ increases sharply over recently opened water (*SI Appendix*, Fig. S4), potentially linked to increases in phytoplankton productivity. Open water produces more sea spray emissions than ice-covered regions. This may also contribute to increased *N*_*d*_, but with limited seasonality. In summary, in situ measurements of aerosol concentrations and aircraft measurements of *N*_*d*_ quantitatively support the seasonal and spatial patterns of satellite-derived *N*_*d*_ in the SO.

### What Does Pristine PD *N*_*d*_ Tell Us about GCM Discrepancies in aci?

We have demonstrated that the PD *N*_*d*_ hemispheric contrast is a useful framework for interpreting GCM behavior. We have also demonstrated that MODIS *N*_*d*_ is a reliable estimate of SH *N*_*d*_ as well as NH *N*_*d*_. We further showed that summertime high-latitude *N*_*d*_ can be a factor of three smaller in GCMs than in satellite estimates (Fig. 1 *A* and *B*). This leaves us with an important question: How can we use the information contained in estimates of PD *N*_*d*_ in pristine regions to understand what processes are currently missing or poorly captured in GCMs? Resolving these discrepancies is important for accurately representing aci occurring in the PD and PI.

**Fig. 3.**
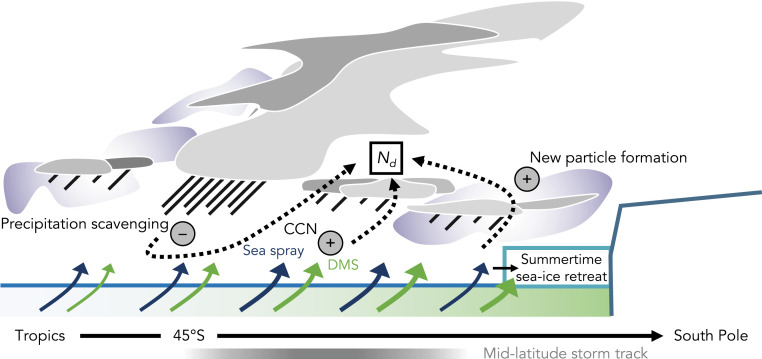
Schematic depicting main sources (+) and sinks (−) of aerosol affecting the cloud droplet number concentration (*N*_*d*_) in the Southern Ocean. Approximate location of the climatological midlatitude storm track is shown for reference.

In answering this question, it is important to remember that the amount of aerosol available to be activated into cloud droplets is a function of both aerosol sources and sinks. The amount of cloud droplets, *N*_*d*_, will also be a function of these sources and sinks. The SO is a pristine environment where aerosol sources are analogous to the PI. However, both aerosol sources and sinks vary across the SO (see diagram in Fig. 3). Sources of SO CCN are a combination of sea spray and biogenic sources and depend upon surface and free-tropospheric physical and chemical processes (67). Emissions from ocean biology also influence NH maritime *N*_*d*_ and are known to control NH marine CCN and nucleation-mode seasonality (35–37). Sea spray emissions in the SH vary a small amount during the year (34, 67) and are unlikely to be contributing to the seasonal cycle of *N*_*d*_ (34). The dominant contributor to biogenic CCN is thought to be DMS emissions from phytoplankton with regional contributions from primary emissions of organically enriched sea spray (34, 68).

It is thought that nucleation and growth of new particles results in between 40% and 70% of global CCN (69). DMS oxidation products along with other stabilizing compounds can act as precursors for gas-to-particle conversion and form new, nucleation-mode aerosols (56, 57). New particle formation has been documented in the free troposphere, coastal regions, at ice edges, near clouds, and in the boundary layer if conditions are favorable (57). Biogenic free tropospheric new particles are known to influence boundary layer CCN in both the NH (36) and across the SH (58, 59). SO air masses observed at the edge of Antarctica have signatures of sulfate-based new particle formation, contributing to high summertime and strongly seasonal nucleation and CCN concentrations (47, 58, 70, 71). In the Antarctic and Arctic, particle formation events are typically connected to emissions from biological activity or iodine emitted from melting ice (57, 71). In the Arctic, where seasonal ice melt increases biological activity initiating bursts of new particle formation, a ∼20% summertime increase in CCN concentration can occur (72). In the Antarctic, a similar summertime increase in new particle formation over the seasonal ice zone occurs (71). Increasing concentrations of new particles during sea ice melt may be contributing to increases in *N*_*d*_ over retreating sea ice (*SI Appendix*, Figs. S3*D* and S4).

The primary sink of CCN is coalescence scavenging associated with the formation of precipitation (73). The rapid decrease in *N*_*d*_ off the coast of Antarctica may be related to enhanced precipitation scavenging associated with midlatitude storms (Fig. 3). This idea is supported by the location of the minima in MODIS *N*_*d*_ coinciding with the climatological position of the SH storm track in austral summer (Fig. 2*A*) (74). In situ measurements of trace gases and aerosol number concentration indicate that the biogenic CCN may be enhanced near Antarctica (55, 58), possibly resulting from a weakening in precipitation scavenging, an increase in biological activity near ice edge, or a combination of both effects.

To assess the role of precipitation sinks in the SO, we apply a simple source and sink budget model for CCN and, equivalently, *N*_*d*_ (73). The ratio between *N*_*d*_ and *N*_*d*_ computed with no precipitation loss is inversely proportional to the precipitation rate and is insensitive to the aerosol source term (see *Materials and Methods*). Unsurprisingly, the strongest precipitation sink is in the heavily precipitating SH storm track (∼50°S; see *N*_*d*_ decrease in Fig. 1*A*), and the budget model shows that coalescence scavenging drives down *N*_*d*_ to ∼30% of the values that would occur without a precipitation sink (exact values shown in *SI Appendix*, Fig. S5 *K*–*O*). Poleward of the storm track, at 65°S, *N*_*d*_ is only reduced to ∼70% of the value without a precipitation sink (*SI Appendix*, Fig. S5*K*). This may be a reflection of marine boundary layer (MBL) depth being shallower over the cold waters near Antarctica and decreasing the clouds’ capacity to support significant boundary layer cloud precipitation (75). The budget model also shows us that the fractional reduction of *N*_*d*_ by precipitation has only a weak seasonal cycle and is therefore not a major determinant of the seasonal *N*_*d*_ cycle over the SO region. This is consistent with the conclusion, from previous studies, that seasonal variability in *N*_*d*_ over the SO is driven primarily by biogenic aerosol sources (33, 34, 53, 76).

Based on our budget model assessment, we conclude that precipitation scavenging acts as a strong sink of CCN in the midlatitude storm track and drives the decrease in *N*_*d*_ equatorward of Antarctica. There is evidence that models precipitate too much in this region, possibly creating too strong a sink of *N*_*d*_ in the storm track in GCMs (77). Discrepancies between satellite estimates and modeled *N*_*d*_ near Antarctica, where precipitation sinks of aerosol are weak, indicate that aerosol production processes are not well represented in GCMs either. It is likely that the same aerosol processes that are important near Antarctica (i.e., those linked to ocean biology) influence midlatitude regions that have stronger precipitation sinks. Missing or incomplete mechanisms for producing CCN in the biologically active region near Antarctica have implications for CCN across the SH. Disagreement between modeled and satellite estimates in summertime midlatitude *N*_*d*_ in the NH, which has similar marine biogenic aerosol sources, suggests that these model discrepancies are not relegated to the SH alone. GCMs may be additionally suffering from equifinality issues (43, 44). Thus, representation of the mechanisms leading to high near-Antarctic and summertime SO *N*_*d*_ as well as more accurate representations of precipitation sinks are important for advancing estimations of *N*_*d*_ in the PI and *N*_*d*_ in PD pristine regions.

What factors could be leading to the GCM underestimations of SO *N*_*d*_? One possibility is that GCMs do not emit enough DMS into the SO, stalling particle formation and growth processes. The amount of DMS in SO seawater and the exchange of DMS between water and air are uncertain (78). Enhancement of global DMS concentrations by 70% in HadGEM3-GA7.0 did not substantially alter SO *N*_*d*_ or the hemispheric contrast, ∆*N*_*d(NH-SH)*_ (Fig. 1 and *SI Appendix*, Fig. S1). Uncertainty in air−sea exchange processes complicates this evaluation (79). However, sensitivity tests in HadGEM3-GA7.1 demonstrate that inclusion of more complete sulfate chemistry processes (relevant for summer CCN) and improved parametrizations of sea salt production (relevant for winter CCN) brings modeled *N*_*d*_ into closer agreement with MODIS *N*_*d*_ in the SO (80). Another possibility is that nucleation of new particles, particularly from DMS oxidation products, is less efficient in GCMs. If natural new particle formation mechanisms are not included in GCMs, then it is likely that models systematically overestimate the strength of aerosol−cloud radiative forcing (81). This would result in an *RF*_*aci*_ that is too strong in models, consistent with our constraint of the PPE members by ∆*N*_*d(NH-SH)*_.

## Discussion

The hemispheric contrast in oceanic *N*_*d*_ (∆*N*_*d(NH-SH)*_) offers a constraint on changes in global mean *N*_*d*_ between the PI and PD (*∆N*_*d(PD-PI)*_) and, by extension, on *RF*_*aci*_ (Fig. 1 *C* and *D*). Based on the satellite-derived ∆*N*_*d(NH-SH)*_ and output from GCMs, *∆N*_*d(PD-PI)*_ is constrained to be between 8 cm^−3^ and 24 cm^−3^. *RF*_*aci*_ is constrained to be between −1.2 W⋅m^−2^ and −0.6 W⋅m^−2^. This constraint on *RF*_*aci*_ agrees with the most probable range of −1.2 W⋅m^−2^ to −0.3 W⋅m^−2^ developed in Bellouin (3). The range developed in Bellouin (3) utilized observational studies relating aerosol variance to *N*_*d*_ variance. Our analysis is insensitive to aerosol observations and provides an important confirmation of this range using a different approach. Our analysis also suggests that the weaker *RF*_*aci*_ in the Bellouin (3) range is not consistent with the *N*_*d*_ calculated from satellite data. However, an important caveat to this study and other studies seeking to offer an observational constraint on GCM behavior using a single criterion is that it may result in an overly tight constraint on model behavior due to structural uncertainties in the GCM (42, 45). Future analysis will combine the hemispheric contrast in *N*_*d*_ with other constraints on model behavior to reinforce its robustness.

A key finding of this study is that models generally simulate larger hemispheric *N*_*d*_ differences than are calculated from satellite retrievals (Fig. 1*C*). Satellite-derived ∆*N*_*d(NH-SH)*_ is relatively low, partly due to high local summertime *N*_*d*_ over the SH midlatitudes (Fig. 1*A*). MODIS *N*_*d*_ near Antarctica was found to be even higher, reaching values close to those in outflows from North America and East Asia. Evaluation of in situ data from cruises, flight campaigns, and stations on the Antarctic continent confirms the accuracy of satellite-derived SO *N*_*d*_ (Fig. 2). Evaluation of SH CCN precipitation sinks demonstrates that high summertime *N*_*d*_ near Antarctica is, in part, due to low removal rates by precipitation scavenging on the poleward flank of the storm track (Fig. 3). None of the GCMs surveyed here or the 235 original PPE ensemble members come near to reproducing the high near-Antarctic values in summertime (Fig. 1; individual models and PPE members shown in *SI Appendix*, Figs. S1 and S2, respectively), suggesting models are missing key processes and/or emission sources important for CCN near Antarctica and potentially across the SH.

Ultimately, *N*_*d*_ is the variable that controls aci in liquid clouds. This quantity is the product of aerosol emissions, removal, transport, processing, and nucleation, and it serves as a key assessment of GCM skill in portraying aci. This reinforces the need to continue to create *N*_*d*_ datasets from new satellites and in new ways. We propose that future evaluations of GCM aci use the information contained within the contrast between pristine and polluted regions as an important test of realism in addition to evaluation of predicted *N*_*d*_ within anthropogenically perturbed regions.

## Materials and Methods

In this paper, we contrast *N*_*d*_ predicted by GCMs with satellite data and in situ observations. *N*_*d*_ is always presented in-cloud, not averaged across cloudy and cloud-free regions. The central remote sensing dataset used in this study is MODIS C5.1 utilizing the 3.7-µm channel (82) during the period 2003–2015 (83). The calculation of *N*_*d*_ from MODIS retrievals of cloud optical depth (*τ*) and cloud droplet effective radius (*r*_*e*_) is not always reliable. The retrieval criteria presented in ref. 84 are used to select for times and places consistent with the assumptions made in the retrievals of *τ* and *r*_*e*_ from satellite radiances as well as in the calculation of *N*_*d*_ resulting from these quantities. Briefly, these criteria are that solar zenith angles are below 65°, cloud tops are within 3.2 km of the surface, and liquid cloud fractions are greater than 80% in a 1° × 1° region (21, 84, 85). Data are filtered using these criteria based on individual level-2 swaths (as opposed to daily averages) that have been averaged to 1° × 1°.

Satellite-derived *N*_*d*_ values in the SO are evaluated using observations from a variety of campaigns and ground stations. In situ CCN observations from McMurdo Station (48) and King Sejong Station (47) are drawn from the reported monthly and seasonal mean values in the literature. MODIS *N*_*d*_ for these regions is shown averaged across a 4° box centered at the respective stations (*SI Appendix*, Fig. S3*A*). RITS total aerosol concentration was obtained from data provided to the Global Aerosol Synthesis and Science Project (GASSP) (86). ORCAS *N*_*d*_ data measured between the surface and 3 km are obtained from the Earth Observing Laboratory (EOL) at the National Center for Atmospheric Research (NCAR) (87). Sea ice cover was interpolated to the ORCAS flight track. The sea ice cover used in this analysis was from the Operational Sea Surface Temperature and Sea Ice Analysis (OSTIA) provided with the Modern-Era Retrospective analysis for Research and Applications, Version 2 (MERRA2) data product (65). The *N*_*d*_ calculated by MODIS is sorted as a function of sea ice vs. open water and compared to similarly separated observations from ORCAS. For the comparison in *SI Appendix*, Fig. S3*D*, MODIS *N*_*d*_ from 2003 to 2015 was restricted to only the days of the year and 1° regions where ORCAS measured *N*_*d*_.

The GCM *N*_*d*_ examined in this study is provided by models participating in the Aerocom phase II indirect experiment (30), sensitivity experiments conducted in the development of HadGEM3-GA7.1 (31), and a PPE within HadGEM3-GA4-UKCA (23). The Aerocom phase II models considered are CAM5 (Community Atmosphere Model), CAM5-CLUBB (Cloud Layers Unified By Binormals), CAM5-MG2, CAM5-CLUBB-MG2, ECHAM6.1.0-HAM2.2, SPRINTARS (Spectral Radiation-Transport Model for Aerosol Species), and SPRINTARSKK (SPRINTARS with Khairoutdinov and Kogan autoconversion scheme). The model variants of the UM (Unified Model) examined in Mulcahy et al. (31) shown here are GA7.0, GA7.1, GA7.0_dms (DMS in sea water set to 170% of climatology), GA7.0_act (changes to the activation scheme), and GA7.0_comb (GA7.1 with no cloud tunings). Following standard experiment protocols for determining the anthropogenic aerosol ERF (88, 89), the model simulations and subsequent change in *N*_*d*_ do not account for climate-driven changes in natural aerosol emissions (e.g., wind speed changes) over the PI to PD period that may influence the *N*_*d*_ concentration in the SO (90).

Multimodel ensembles (such as Aerocom) are invaluable for quantifying the magnitude of differences between models due to choices of physical process representations. However, this type of ensemble neglects the uncertainty within individual models. PPEs provide a useful means of quantifying single-model uncertainty (91); however, they neglect uncertainty caused by particular choices of process representations. We represent single-model uncertainty using output from a PPE of the HadGEM-GA4-UKCA GCM. In this PPE, 26 aerosol process, emission, and deposition parameters were simultaneously perturbed, allowing for assessment of a broad range of model behavior (*SI Appendix*, Fig. S2). The PPE contains 235 model variants, each with a unique combination of the parameter values. Each PPE member simulated PD *N*_*d*_ resolved in space and time, PI global mean *N*_*d*_, and top-of-the-atmosphere radiative fluxes (used to calculate *RF*_*aci*_). Horizontal winds and temperature fields were relaxed (92) toward 2008 meteorology from the European Centre for Medium-Range Weather Forecasts (ECMWF) Re-Analysis (ERA) ERA-Interim and forced with year 2008 anthropogenic aerosol emissions from the MACCity emission inventory (93). To quantify uncertainty in changes over the industrial period, each of the 235 simulations has a partner simulation with identical parameter values, but with anthropogenic emissions from 1850 prescribed instead of PD emissions. The model was configured so that the first indirect effect of aerosols can be quantified in the absence of aerosol−cloud adjustments. Variations in *N*_*d*_ over the ensemble are caused entirely by differences in aerosol size distributions due to combinations of the 26 parameter values. We use the 235-member PPE to build statistical emulators of *N*_*d*_ and *RF*_*aci*_. A sample of 1 million model variants (parameter combinations) is drawn from the emulator for each variable (32). Creation of the emulator assumes trapezoidal priors developed using expert solicitation (23). This makes the sample members more centralized in the multidimensional parameter space compared to the uniform priors assumed in earlier works (42, 45, 46, 94). If these uniform priors, which assume the entire range for all parameters are equally likely, are used in our analysis instead, the range of possible *RF*_*aci*_ consistent with estimated ∆*N*_*d(NH-SH)*_ is between −1.4 W⋅m^−2^ and −0.5 W⋅m^−2^ (*SI Appendix*, Fig. S6).

Hemispheric contrast in *N*_*d*_ (∆*N*_*d(NH-SH)*_) is calculated as the difference in the annual mean of the area-weighted *N*_*d*_ concentrations over oceans 30°N to 60°N and 30°S to 60°S. *N*_*d*_ data in months and latitudes where MODIS retrievals are unavailable are removed from the GCM data before calculating the hemispheric contrast, to avoid biases in comparing estimates from MODIS and modeled *N*_*d*_. The region 30°S to 30°N is excluded because the retrieval of *N*_*d*_ by MODIS in convection is less robust (21). The random uncertainty in *N*_*d*_ calculated from MODIS is relatively small once it is averaged across a 30°-latitude band (21). The 95% confidence on the hemispheric contrast was calculated by taking the SE in the annual hemispheric contrast in the years 2003–2015 and assuming a normal distribution. Evaluation of *N*_*d*_ calculated from MODIS data has shown small systematic error (21). Further, by examining the difference between hemispheres, we expect any systematic bias in the MODIS retrievals will be reduced.

The best-fit line relating ∆*N*_*d(NH-SH)*_ to *∆N*_*d(PD-PI)*_ and *RF*_*aci*_ was calculated using least-squares regression on the PPE sample members. The prediction band on the best-fit line was used to quantify the possible range of *N*_*d*_ and *RF*_*aci*_ because all PPE sample members are considered to be equally valid representations of the real world. The prediction band about the best-fit line was calculated by fitting the 95th percentile of PPE members in 30 quantiles of hemispheric contrast.

To quantitatively estimate the impact of MBL precipitation on the seasonal climatology of *N*_*d*_ over the SO, we use the source and sink aerosol budget model developed in ref. 73. The model was developed for use over those parts of the global oceans where CCN concentration loss rates are driven primarily by coalescence scavenging in MBL cloud systems (95). Modeled mean MBL CCN estimates from the model appropriate for describing the monthly mean climatology of *N*_*d*_ were shown to agree well with the observed *N*_*d*_ off the coast of Chile (73) and between California and Hawaii (96).

We apply the ref. 73 model to estimate the impact of MBL cloud precipitation on the summertime meridional gradient of *N*_*d*_ over the SO. Using the equilibrium number concentration from the model (ref. 73, equation **2**), we construct a ratio between *N*_*d*_ with precipitation loss and *N*_*d*_ computed with no precipitation loss. This is found to be inversely proportional to the precipitation rate,

$$\frac{N_{d}\left(precip\right)}{N_{d}\left(no\;precip\right)}={\left(1+\frac{hKP_{CB}}{Dz_{i}}\right)}^{-1}.$$ [1]

As in ref. 73, *h* is the cloud thickness (derived from MODIS LWP using the adiabatic assumption), *K* is a constant (97), *P*_*CB*_ is cloud base precipitation rate derived from CloudSat (73), *D* is the surface divergence for low cloud scenes, and *z*_*i*_ is the planetary boundary layer depth. Because of the difficulty of isolating surface divergence for low cloud scenes over the midlatitude storm tracks, we note that D*z_*i*_ is the subsidence rate at cloud top, which we assume to be equal to the entrainment rate, which we estimate as 4 mm/s, consistent with typical values found in low clouds in the subtropics and midlatitudes. We estimate the coalescence scavenging sink using the CloudSat-derived precipitation rate product (98). This product attempts to estimate precipitation from all cloud systems, not only those arising from MBL clouds.

Previous applications of this model examined the eastern ocean subtropical systems (73, 96) where precipitation was primarily derived from low cloud systems. In contrast, across the SO, there is considerably more precipitation emanating from deeper precipitating systems (99). This is accounted for by only considering CloudSat precipitation estimates with detectable echo tops below 3 km altitude. This attempts to ensure that only precipitation that has a significant contribution to the coalescence scavenging of MBL CCN is used as input to the CCN and *N*_*d*_ budget models. This choice is based on data from the Azores, which straddles the boundary between the subtropics and the midlatitudes (∼40°N), showing that between 15% and 30% of all precipitation reaching the surface originated from clouds with tops below 3 km (100). Similarly, between 30°S and 70°S, with weak dependence on latitude, we find that 15 to 35% of all precipitation reaching the surface originates from clouds with tops below ∼3 km (*SI Appendix*, Fig. S5 *A*–*E*).

## Supplementary Material

Supplementary File

## Data Availability

The observational and remote sensing datasets supporting this analysis are available either in previously published works or at supporting websites. This includes the central dataset in our analysis, the multiyear MODIS *N*_*d*_ product which is hosted at the Centre for Environmental Data Analysis (83). All relevant citations and supporting sites are noted in the descriptions of these datasets in *Materials and Methods*. Aerocom model simulations are similarly available from their support website (https://aerocom.met.no). Raw simulation output data from the HadGEM-UKCA PPE ensembles are available from the JASMIN (Joint Analysis System Meeting Infrastructure Needs) data infrastructure (www.jasmin.ac.uk). Some of the climate‐relevant fields are derived and stored for all ensemble members and made available as a community research tool.
