# Supplementary material for: The hemispheric contrast in cloud microphysical properties constrains aerosol forcing
Source: Proc Natl Acad Sci U S A. 2020 Jul 27;117(32):18998–9006. doi: 10.1073/pnas.1922502117 (PMC7431023; doi:10.1073/pnas.1922502117)
Supplement: Supplementary File [file pnas.1922502117.sapp.pdf]

PNAS
www.pnas.org

Supporting Information for

The hemispheric contrast in cloud microphysical properties constrains aerosol forcing

Isabel L. McCoy [a,1,2], Daniel T. McCoy [b,1,3], Robert Wood [a], Leighton Regayre [b], Duncan Watson-Parris [c], Daniel P. Grosvenor [b,d], Jane P. Mulcahy [e], Yongxiang Hu [f], Frida A.-M. Bender [g,h], Paul R. Field [b,e], Kenneth S. Carslaw [b], Hamish Gordon [b,i]

[a] Atmospheric Sciences Department, University of Washington, Seattle, WA 98105;
[b] Institute for Climate and Atmospheric Science, School of Earth and Environment, University of Leeds, LS2 9JT Leeds, United Kingdom;
[c] Department of Physics, University of Oxford, Oxford OX1 3PU, United Kingdom;
[d] National Centre for Atmospheric Science, University of Leeds, LS2 9JT Leeds, United Kingdom;
[e] Met Office, Exeter EX1 3PB, United Kingdom;
[f] Atmospheric Composition Branch, NASA Langley Research Center, Hampton, VA 23681;
[g] Department of Meteorology, Stockholm University, SE-106 91 Stockholm, Sweden;
[h] Bolin Centre for Climate Research, Stockholm University, SE-106 91 Stockholm, Sweden;
[i] College of Engineering, Carnegie-Mellon University, Pittsburgh, PA 15213

[2] To whom correspondence may be addressed. Email: imccoy@uw.edu.

[1] I.L.M. and D.T.M. contributed equally to this work.
[3] Present address: Department of Atmospheric Science, University of Wyoming, Laramie, WY 82071.

This PDF file includes:

Fig. S1 to S6
References

www.pnas.org/cgi/doi/10.1073/pnas.1922502117

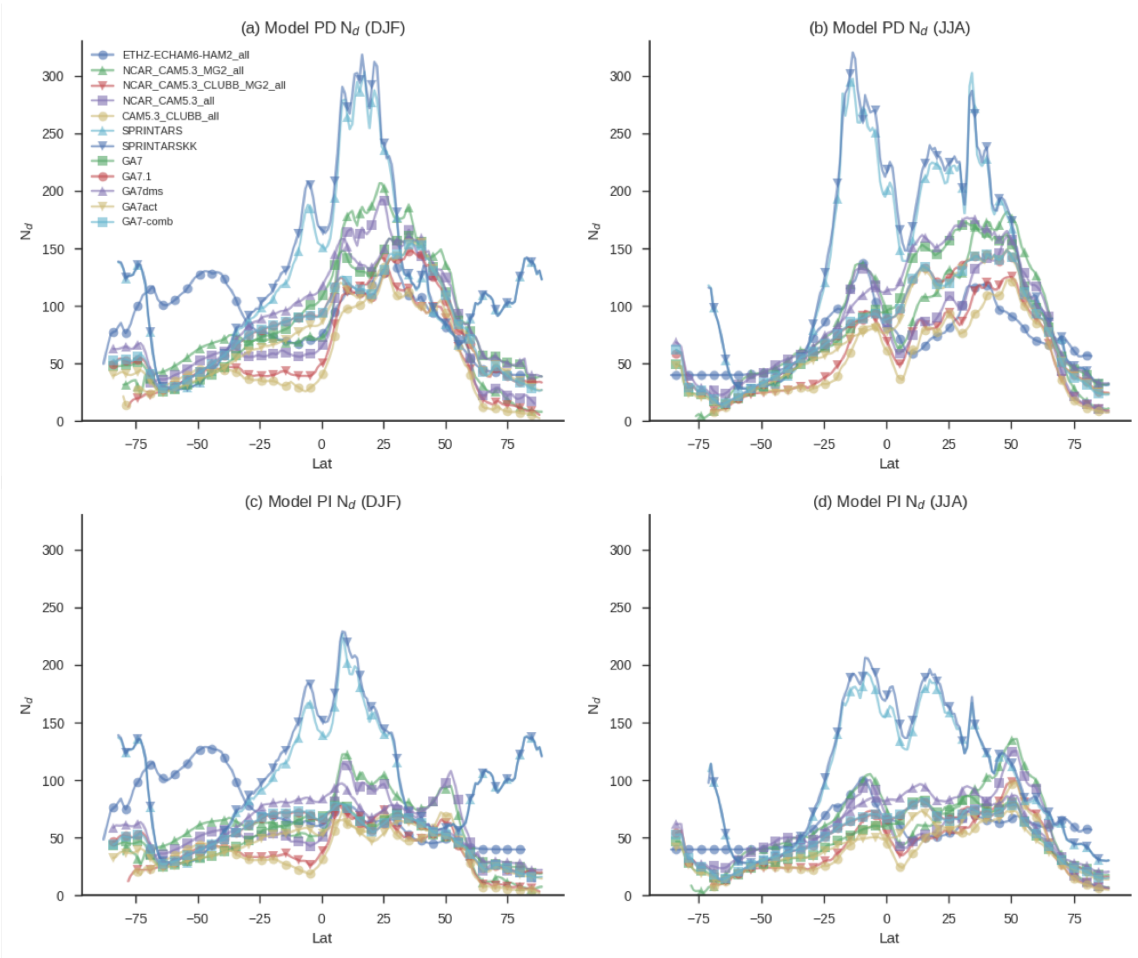

Fig. S1. Oceanic Present Day (a, b) and Pre-Industrial (c, d) seasonal mean N_d from Aerocom-II models (1) and HadGEM3-GA7.0 and GA7.1 (2) models for DJF (a, c) and JJA (b, d).

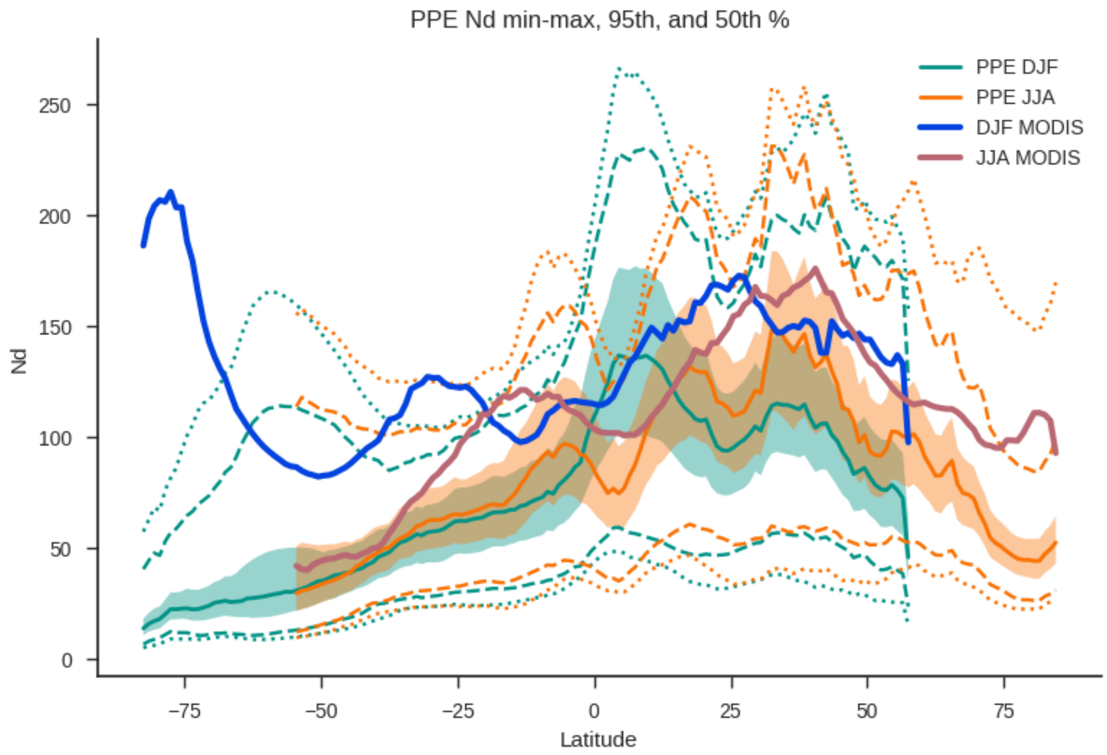

Fig. S2. Zonal-mean oceanic PD N_d from PPE model members (3). The median (solid lines), 50th (shading) and 95th percentile (dashed lines), and minimum and maximum (dotted lines) of all ensemble members are shown for DJF and JJA. MODIS observations for DJF and JJA are shown for reference (4). Geographically resolved PI N_d was not archived from the PPE at the time of integration.

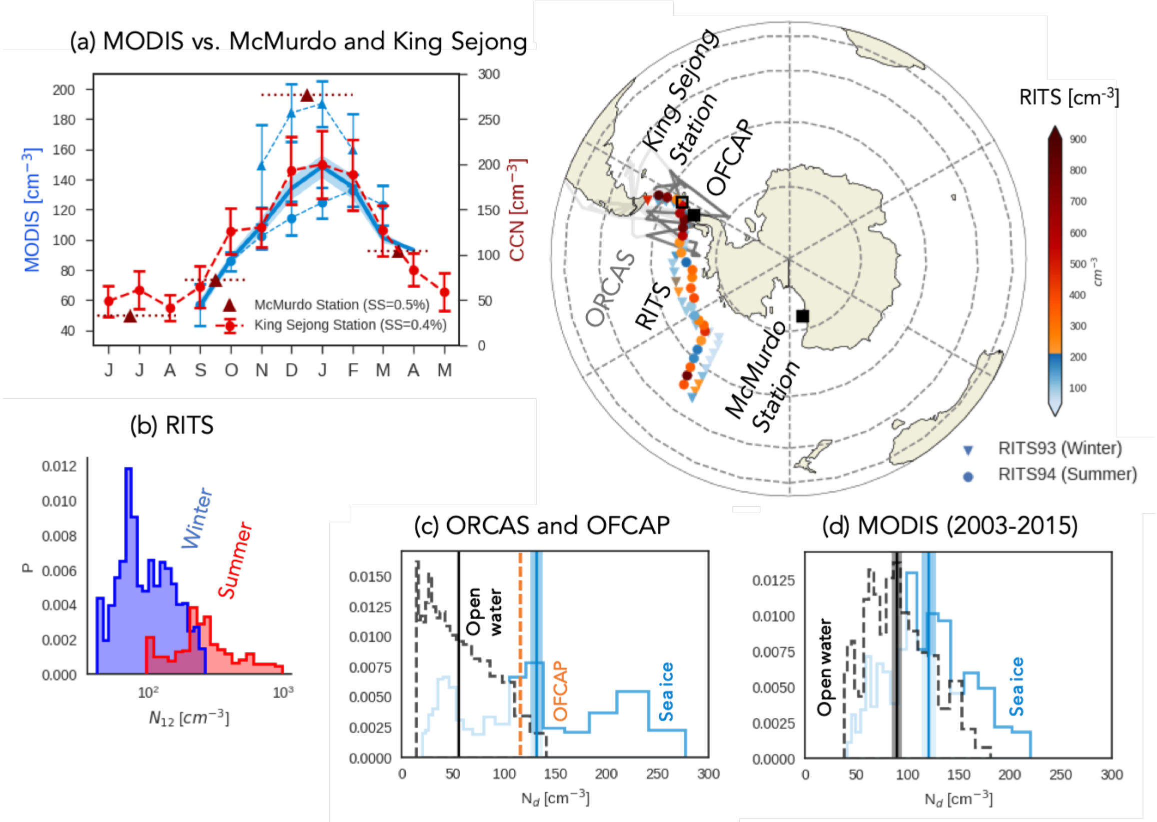

Fig. S3. Map of Southern Ocean field campaign locations with colors marking RITS (5, 6) condensation nuclei (aerosol diameter > 12 nm) from Winter (triangles) and Summer (circles). Locations of the OFCAP (7) and ORCAS (8) campaigns, McMurdo Station (9), and King Sejong Station (10) are also marked. (a) Seasonal cloud condensation nuclei concentrations from McMurdo Station (red triangle, 0.5% saturation) and King Sejong Station (red dots, 0.4% saturation). MODIS N_d poleward of 60°S (solid blue line) and in 4° boxes centered at McMurdo Station (blue triangles) and King Sejong Station (blue dots). The interannual standard deviation in the monthly mean MODIS N_d is shown as shading for the zonal mean poleward of 60°S and as error bars for station means. (b) PDF for RITS Winter '93 (blue), Summer '94 (red) CN concentration as in map. (c) PDFs of summertime N_d observations from ORCAS which have been separated into observations taken where some sea ice is present (blue) and over ice-free water (grey) based on OSTIA sea ice extent (11) interpolated to the flight track. Mean values are shown with vertical lines. The OFCAP campaign mean N_d is shown for reference. (d) Summertime MODIS N_d from 2003-2015 following the ORCAS flight track are shown separated by OSTIA sea ice extent as in (c).

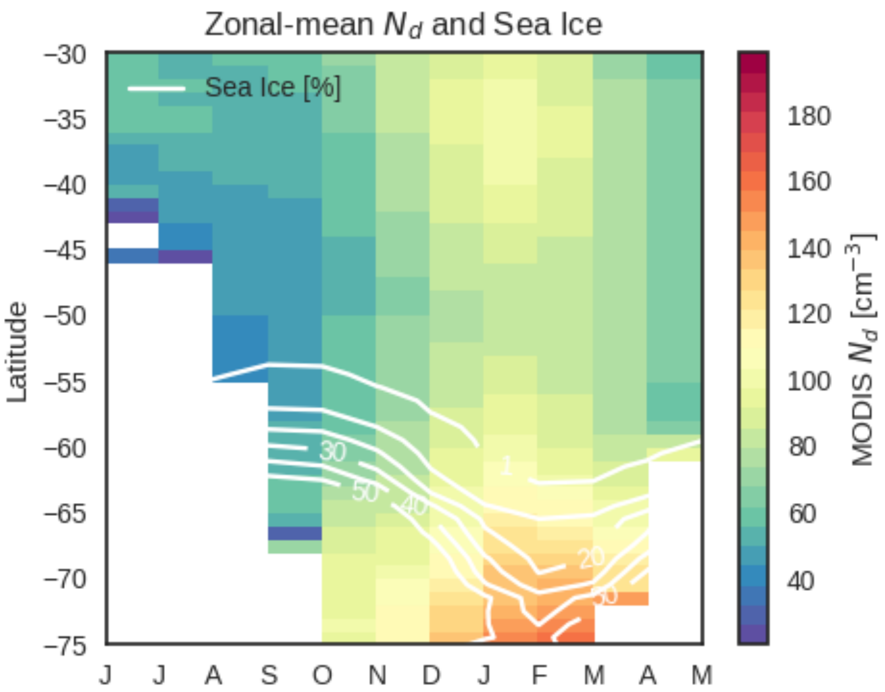

Fig. S4. Seasonal cycle of Southern Ocean N_d from MODIS (colors) with OSTIA sea ice extent (white lines) included for reference. Data has been masked to remove land.

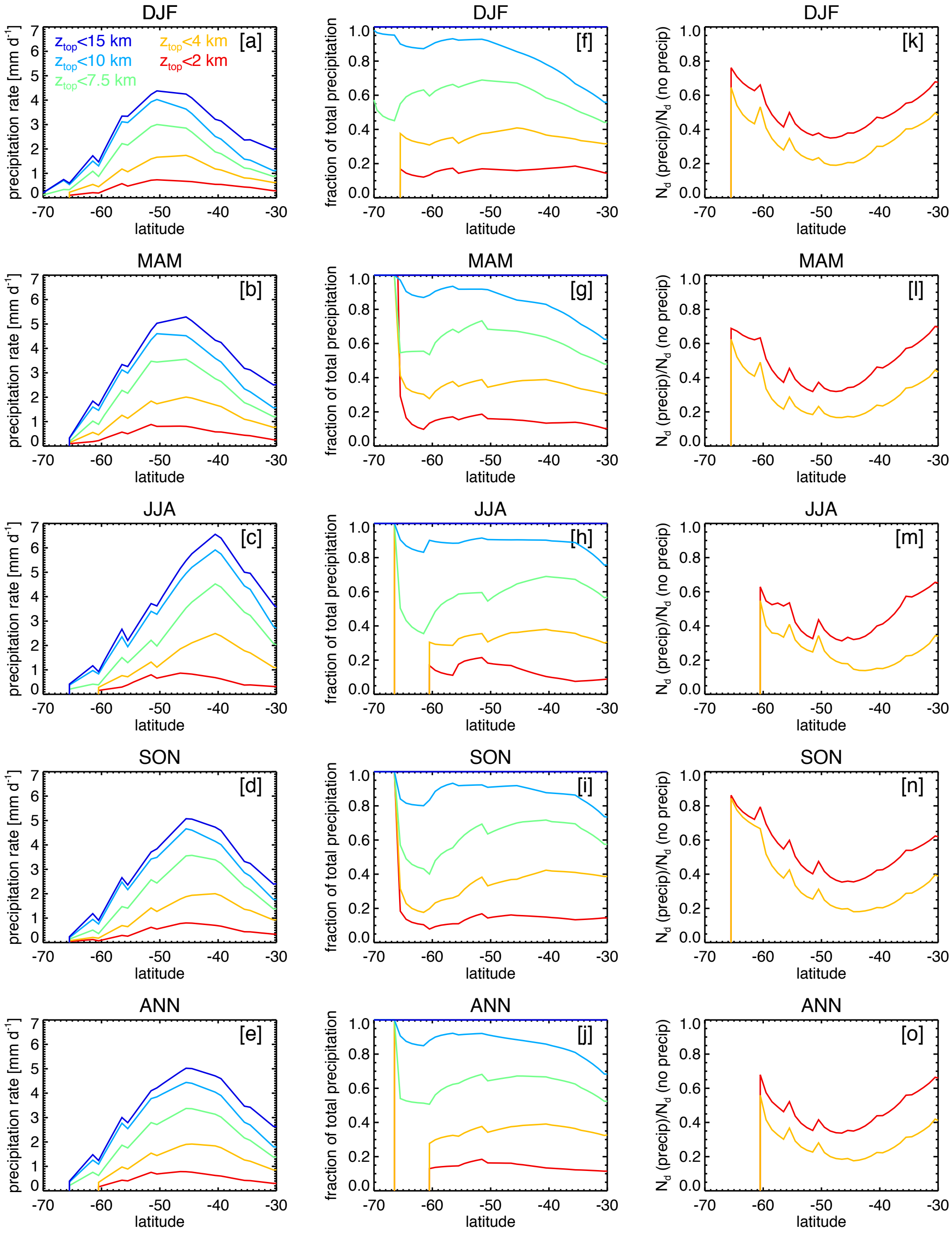

Fig. S5. Seasonal (a-d) and annual (e) zonal mean area-averaged precipitation rates (12) emanating from columns with maximum echo top heights lower than the color-coded values indicated on panel (a). Panels (f-j) show the same information but as a fraction of the total precipitation, with color coding the same as in panels (a-e). Panels (k-o) show the MBL CCN and N_d budget model (13) estimates of the relative suppression of N_d by coalescence scavenging (detailed in methods), using precipitation with echo tops below either 2 km (red) or 4 km (yellow) as a means to bound the expected impact of low cloud precipitation on N_d.

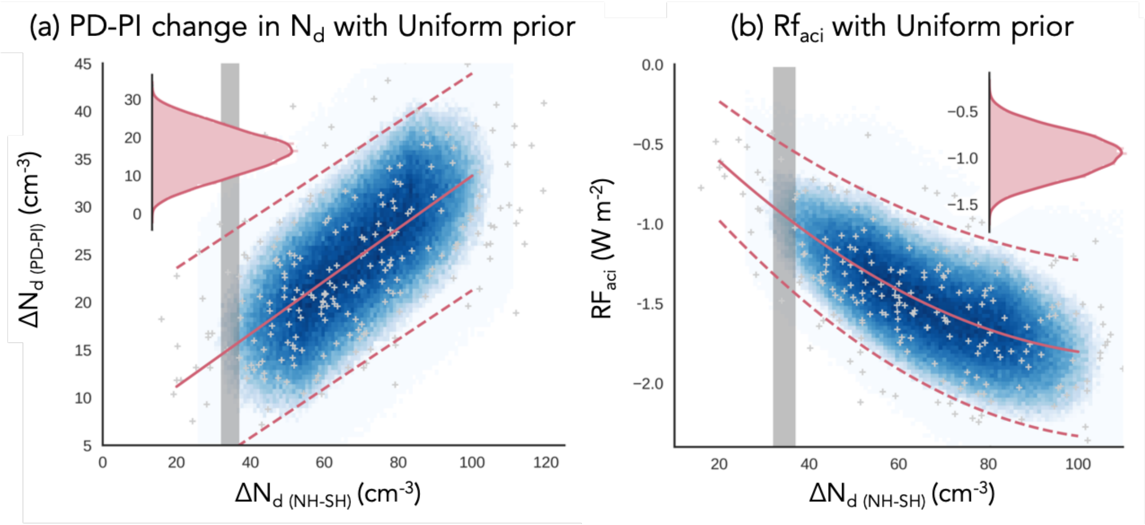

Fig. S6. As in Fig. 1*B, C* but assuming uniform pdf priors (14-17) to generate the PPE sample members (blue shading) from the individual model members (white crosses) (18). (Insets) The PDF of the emulated PPE member values within the observationally constrained range of $\Delta N_{d(NH\text{-}SH)}$ (gray) is shown in the top left for $\Delta N_{d(PD\text{-}PI)}$ (a) and top right for RF_{aci} (b).

References

1. S. Ghan *et al.*, Challenges in constraining anthropogenic aerosol effects on cloud radiative forcing using present-day spatiotemporal variability. *Proceedings of the National Academy of Sciences* 10.1073/pnas.1514036113 (2016).
2. J. P. Mulcahy *et al.*, Improved Aerosol Processes and Effective Radiative Forcing in HadGEM3 and UKESM1. *J. Adv. Model. Earth Syst.* **10**, 2786-2805 (2018).
3. M. Yoshioka *et al.*, Ensembles of Global Climate Model Variants Designed for the Quantification and Constraint of Uncertainty in Aerosols and Their Radiative Forcing. *J. Adv. Model. Earth Syst.* 10.1029/2019ms001628 (2019).
4. D. P. W. Grosvenor, R., Daily MODIS (MODerate Imaging Spectroradiometer) derived cloud droplet number concentration global dataset for 2003-2015. Centre for Environmental Data Analysis. http://catalogue.ceda.ac.uk/uuid/cf97ccc802d348ec8a3b6f2995dfbbff.
5. D. S. Covert, V. N. Kapustin, T. S. Bates, P. K. Quinn, Physical properties of marine boundary layer aerosol particles of the mid-Pacific in relation to sources and meteorological transport. **101**, 6919-6930 (1996).
6. P. K. Quinn, V. N. Kapustin, T. S. Bates, D. S. Covert, Chemical and optical properties of marine boundary layer aerosol particles of the mid-Pacific in relation to sources and meteorological transport. *Journal of Geophysical Research: Atmospheres* **101**, 6931-6951 (1996).
7. T. Lachlan-Cope, C. Listowski, S. Shea, The microphysics of clouds over the Antarctic Peninsula Part 1: Observations. *Atmospheric Chemistry and Physics* **16**, 15605-15617 (2016).
8. B. B. Stephens *et al.*, The O2/N2 Ratio and CO2 Airborne Southern Ocean Study. *Bulletin of the American Meteorological Society* **99**, 381-402 (2018).
9. J. Liu *et al.*, High summertime aerosol organic functional group concentrations from marine and seabird sources at Ross Island, Antarctica, during AWARE. *Atmospheric Chemistry and Physics* **18**, 8571-8587 (2018).
10. J. Kim *et al.*, Seasonal variations in physical characteristics of aerosol particles at the King Sejong Station, Antarctic Peninsula. *Atmospheric Chemistry and Physics* **17**, 12985-12999 (2017).

11. C. J. Donlon *et al.*, The Operational Sea Surface Temperature and Sea Ice Analysis (OSTIA) system. *Remote Sensing of Environment* **116**, 140-158 (2012).
12. J. M. Haynes *et al.*, Rainfall retrieval over the ocean with spaceborne W-band radar. *Journal of Geophysical Research: Atmospheres* **114** (2009).
13. R. Wood, D. Leon, M. Lebsock, J. Snider, A. D. Clarke, Precipitation driving of droplet concentration variability in marine low clouds. *Journal of Geophysical Research: Atmospheres* **117**, n/a-n/a (2012).
14. J. S. Johnson *et al.*, The importance of comprehensive parameter sampling and multiple observations for robust constraint of aerosol radiative forcing. *Atmospheric Chemistry and Physics* **18**, 13031-13053 (2018).
15. L. A. Regayre *et al.*, Aerosol and physical atmosphere model parameters are both important sources of uncertainty in aerosol ERF. *Atmospheric Chemistry and Physics* **18**, 9975-10006 (2018).
16. L. A. Regayre *et al.*, The value of remote marine aerosol measurements for constraining radiative forcing uncertainty. *Atmos. Chem. Phys. Discuss.* **2019**, 1-11 (2019).
17. J. S. Johnson *et al.*, Robust observational constraint of uncertain aerosol processes and emissions in a climate model and the effect on aerosol radiative forcing. *Atmos. Chem. Phys. Discuss.* **2019**, 1-51 (2019).
18. D. Watson-Parris *et al.*, Constraining uncertainty in aerosol direct forcing. *Geophysical Research Letters* 10.1029/2020gl087141 (2020).
